# Exploring Parental Responses to Pre-schoolers’ “Everyday” Pain Experiences Through Electronic Diary and Ecological Momentary Assessment Methodologies

**DOI:** 10.3389/fpsyg.2021.741963

**Published:** 2021-11-04

**Authors:** Grace O’Sullivan, Brian McGuire, Michelle Roche, Line Caes

**Affiliations:** ^1^Centre for Pain Research, National University of Ireland Galway, Galway, Ireland; ^2^School of Psychology, NUI Galway, Galway, Ireland; ^3^Department of Physiology, School of Medicine, NUI Galway, Galway, Ireland; ^4^Galway Neuroscience Centre, NUI Galway, Galway, Ireland; ^5^Division of Psychology, Faculty of Natural Sciences, University of Stirling, Stirling, United Kingdom

**Keywords:** everyday pain, home, digital health, diary, parent, child

## Abstract

**Objective:** Parental influence during children’s “everyday” pain events is under-explored, compared to clinical or experimental pains. We trialed two digital reporting methods for parents to record the real-world context surrounding their child’s everyday pain events within the family home.

**Methods:** Parents (*N* = 21) completed a structured e-diary for 14 days, reporting on one pain event experienced by their child (aged 2.5–6 years) each day, and describing child pain responses, parental supervision, parental estimates of pain severity and intensity, and parental catastrophizing, distress, and behavioral responses. During the same 2-week period, a subsample of parent-child pairs (*N* = 9) completed digital ecological momentary assessments (EMA), immediately after any chosen pain event. Children reported their current pain while parents estimated the child’s pain and indicated their own distress.

**Results:** “Everyday” pain events frequently featured minor injuries to the child’s head, hands or knees, and child responses included crying and non-verbal comments (e.g., “Ouch!”). Pain events occurred less frequently when parents had been supervising their child, and supervising parents reported lower levels of worry and anxiety than non-supervising parents. Child sex was significantly associated with parental estimates of pain intensity, with parents of girls giving higher estimates than parents of boys. Child age was significantly associated with both the number of pain events and with parental estimates of pain intensity and child distress: the youngest children (2–3 years) experienced the fewest pain events but received higher pain and distress estimates from parents than older children. Hierarchal Linear Modeling revealed that parental estimates of pain severity were significant positive predictors of parental distress and catastrophizing in response to a specific pain event. Furthermore, higher levels of parental catastrophic thinking in response to a specific pain event resulted in increased distress, solicitousness, and coping-promoting behaviors in parents. The EMA data revealed that children reported significantly higher pain intensity than their parents.

**Conclusion:** The electronic pain diary provided a key insight into the nature of “everyday” pain experiences around the family home. Digital daily reporting of how the family copes with “everyday” events represents a viable means to explore a child’s everyday pains without disrupting their home environment.

## Introduction

Clinical and experimental literature has demonstrated that parent responses to their child’s pain can positively or negatively influence their child’s responses, impacting outcomes such as child distress, pain sensitivity, and pain tolerance (see Piira et al., 2005; Campbell et al., 2017). The predominant evidence for parental support during their child’s painful experiences has been drawn from clinical (e.g., needle-pain procedures) and experimental literature (e.g., cold-pressor tests or hypothetical pain scenarios). However, clinical procedures do not cover the entire spectrum of experiences that children have with pain. “Everyday” pain events are common experiences for young children, leading to minor injuries such as bumps, bruises, or scrapes (Fearon et al., 1996). Children experience “everyday” pains more often than any other type of pain, with one event occurring approximately every 3 waking hours (Harbeck and Peterson, 1992; Fearon et al., 1996). These experiences are influenced by environmental factors including who is present and how they respond (Fearon et al., 1996; von Baeyer et al., 1998; Noel et al., 2018). Unlike clinical or experimental pain events, everyday pains often require no treatment, and so parents or caregivers typically provide comfort such as asking their child about the pain and offering solutions such as instructing their child to take deep breaths, or using water to “wash the pain away” (Power et al., 2007; Hadjistavropoulos et al., 2011). As parents are present for many of their child’s everyday pain events, it is reasonable to assume that such events present opportunities for understanding how parents and children manage minor childhood pains. However, in comparison to clinical literature, research on everyday pain experiences is scarce.

The paucity of data on “everyday” pains might have resulted partly from difficulties in capturing such pains, as everyday pain events are spontaneous and impossible to replicate faithfully in a laboratory setting (Fearon et al., 1996). Equally, child self-reports of pain might have reliability challenges (Chambers et al., 2002; Emmott et al., 2017), and ARE heavily dependent on both the still-developing cognitive capabilities of the child (von Baeyer et al., 2017) and the social context in which the pain occurs (von Baeyer, 2009). As such, observational methods present the best means to capture the *context* surrounding everyday pain events, having been previously employed in day care centers (Fearon et al., 1996), play activity centers (Noel et al., 2018), and family homes (O’Sullivan et al., 2019). Each of these studies utilized behavioral checklists, and two also attempted to use audio-visual recordings, though not without difficulties. Within an activity center setting, large numbers of parent-child pairs were hard to observe reliably and video-camera footage was of poor quality (Noel et al., 2018). Within family homes, the presence of cameras and researchers increased child distress and parental discomfort (O’Sullivan et al., 2019). Thus, alternative measures are still needed to reliably observe everyday pain events without impacting the natural behavior of families.

Electronic diaries can capture parent insights into pain events in real-time, without intrusion from researchers or recording equipment. The data is recorded instantaneously, reducing the biased recall or insufficient detail associated with retrospective accounts (Palermo et al., 2004). End-of-day pain diaries are widely used and accepted in clinical practice for children and parents to record daily fluctuations in pain intensity and monitor recurrent pains such as persistent headaches or juvenile chronic pain (Gaertner et al., 2004; Palermo et al., 2004; Stinson et al., 2006). Similarly, ecological momentary assessment (EMA) can instantly capture “in-the-moment” reflections about pain intensity, emotional changes, or sleep disturbances (May et al., 2018). However, neither method has previously been utilized beyond clinical settings, to gather data on minor pains. If used in conjunction, parents and children could immediately and accurately capture the context surrounding everyday pains. This could provide valuable insight into bidirectional influences between caregiver and child in the early years. Furthermore, diary methods can explore deviations between *anticipated* and *actual* parental thoughts and behaviors in response to their child’s pain experiences. For instance, parents who report high levels of catastrophizing experience more distress and attempting to curtail their child’s activities to prevent further pain (Caes et al., 2011, 2012). However, variations have been found between parents’ anticipated (trait) and actual (state) levels of catastrophic thinking about their child’s pain, with state levels of catastrophic thinking found to be more strongly associated with parent distress than trait levels (Durand et al., 2017). Repeated measurements of parent behaviors in everyday situations are critical to furthering our understanding of the differences in how parents expect they will respond versus how they respond “in the moment” (Goubert et al., 2012).

Finally, the comparative lack of research into everyday pains has highlighted that, while the potential array of “everyday” pains is vast it is unclear how parents devise their personal taxonomy of pain events. Parents often have to make snap judgments on pain intensity or severity, and their responses are likely to be moderated by a variety of contextual factors (von Baeyer, 2009), including their child’s age or sex, or the level of supervision. Drawing from childhood injury literature, active parental supervision of pre-schoolers is the most effective means to avoid injuries and hazards (Morrongiello et al., 2004) while passive/absent supervision is associated with reduced capacity to intervene in a timely manner, leading to increased frequency and increased severity of injuries (Peterson et al., 2002). Parental supervision strategies vary based on child sex and age, with parents of boys employing more effort-intensive strategies to prevent injuries compared to parents of girls (Morrongiello et al., 2004), and parents monitoring the activities of younger children more closely than older children, with further decreased supervision time as children age (Pollack-Nelson and Drago, 2002). However, little is known about the role of contextual factors, such as parental supervision in understanding parental responses toward their child during everyday pain events.

Consequently, the aims of this study were to (1) capture the context of “everyday” childhood pain events occurring within the family home, using a novel electronic self-report diary measure completed by their parents; (2) trial the use of EMA to determine whether parent and child estimates of everyday pain differ from each other; (3) explore the association between parents expected and actual responses during their child’s pain experiences; and (4) explore whether parental behaviors are influenced by additional contextual factors.

## Methods

All study procedures and materials were granted ethical approval by the University Research Ethics Committee at NUI Galway (Galway, Ireland).

### Participants

**FIGURE 1:**
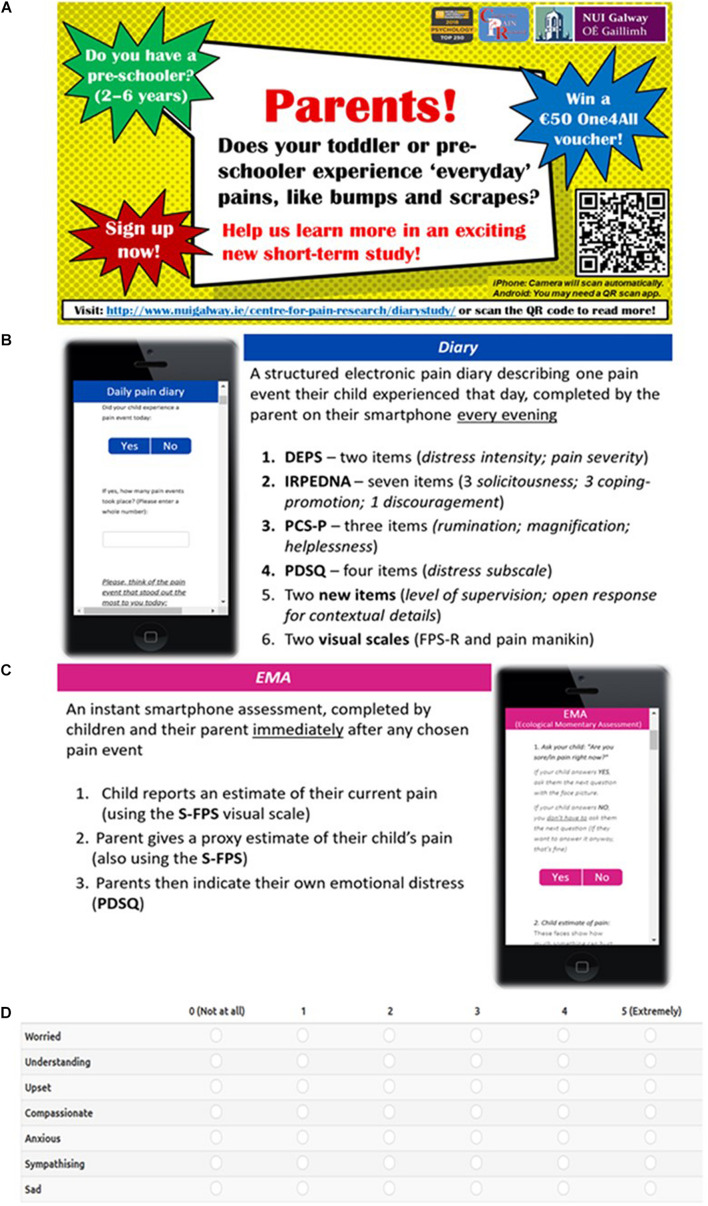
Participant materials: **(A)** Recruitment flyer; **(B)** Overview of diary completion process; **(C)** Overview of EMA completion process; **(D)** Example of the PDSQ scoring scale as viewed on-screen during the EMA.

Recruitment commenced in September 2018 and completed in February 2020. Families with a child between 2.5 and 6-year-olds were recruited through posters and flyers circulated to local child-care centers, playgroups, and activity centers (see Figure 1A); national and local media outlets (radio, newspaper, and social media adverts); and through a study-specific website, which outlined the purpose of the study alongside a participant information sheet. Interested parents contacted the research team via email or social media messaging, and a member of the research team completed eligibility screening. Eligibility criteria included: (1) at least one child aged 2.5–6 years; (2) parent and child are both generally pain-free and healthy (i.e., parent confirmed that neither they nor their child experienced chronic pain or associated health conditions); and (3) parent can read and write in English. Once eligibility had been confirmed, the parent was sent a link to the first stage of the study, where they would complete demographics and pre-diary questionnaires. Following this, the parent would receive an automated email containing a link to the diary, with instructions on how to complete the first diary entry.

A G^∗^Power analysis indicated that a sample of 24 parents would be required for diary completion (0.8 power and 0.5 effect size) (Faul et al., 2007). A total of 40 parents completed the demographics and pre-diary questionnaires. Of these, 21 parents participated in the diary part of the study (52.5%). The remaining 19 parents did not complete the first diary entry following completion of demographics and pre-diary questionnaires. A follow-up reminder email was sent to each participant, but no replies were received to indicate reasons for attrition, and the submitted pre-study data from those 19 parents were removed from further analysis. Of the 21 participating parents, most listed themselves as the child’s biological mother (*N* = 20; 95.2%) with one parent listed as the biological father (*N* = 1; 4.8%) (Table 1). The average parental age was 39.24 years (*SD* = 4.77; range: 29–47 years). The child sample contained 8 girls and 13 boys, and the average age was 3.67 years (44.24 months) (*SD* = 0.98 years; range: 2.25–5.50 years). Most parents were married (81%), and 11 families (52.4%) had more than one child (*M* = 1.95 children; range = 1–5 children). Parents with more than one child in the target age range were informed that they could participate separately with each child if they wished, or if preferred, could choose one eligible child to participate with. Ultimately, no parent participated with more than one of their children, thereby preserving the assumption of data independence. In addition to the diary, nine parent-child pairs elected to complete an EMA during the same period (42.9% of the participants). The nine parents listed themselves as the child’s biological mother (*N* = 8) or biological father (*N* = 1); the average parental age in this subsample was 39.89 years (*SD* = 5.71). The children were four girls and five boys, with an average age of 3.51 years (*M* = 42.11 months; *SD* = 0.88 years).

**TABLE 1:** Demographic information for participating parents and children.

	*N*	Percentage (%)
**Parent**		
Mother	20	95.2
Father	1	4.8
Mean age	39.24 years (*SD* = 4.77 y)	
**Child**		
Female	8	38.1
Male	13	61.9
Mean age	3.67 years *(SD* = 0.98 y)	
Mean children per family	1.95 (range: 1–5)	
**Country of residence**		
Ireland		90.5
United Kingdom		4.8
Canada		4.8
**Marital status**		
Married		81
Single/unmarried		14.3
Other		4.8
**Level of education**		
Bachelor’s degree		38.1
Master’s degree		19
Ph.D.		4.8
Other (e.g., diploma and certificate)		33.3

### Procedure

Once eligibility was confirmed, participants proceeded to the study website to begin participation. This site presented the full information brief, the informed consent brief, a demographic survey, pre-diary questionnaires, and external links to both the diary and the EMA assessments. Participants were invited to bookmark the links for easy access during the study.

All materials from this study were hosted electronically on LimeSurvey (Hamburg, Germany), a *General Data Protection Regulation* (GDPR)-compliant survey package per the University’s ethics policies on data collection and retention. A pre-testing phase took place prior to recruitment, to test the materials and ensure usability. During this phase, a sample of three adults completed the eligibility screening to ensure that sign-up links would be emailed correctly. They then completed the demographics, pre-diary questionnaires, and one diary entry and EMA each, to ensure that the questions and rating scales displayed correctly on different devices (for example, if the diary page would auto-rotate to landscape to complete ratings more easily on an 11-point scale), and typographical errors were corrected. The research team then ensured that data was uploaded correctly and accessible by them on LimeSurvey. All pre-testing data was cleared from the dataset before recruitment opened to participants. Paper copies of all materials were also available upon request in case parents had no access to a smartphone or computer to complete the surveys electronically. Consent and demographic information were requested and stored separately from other data. Participation was anonymized, though an email address was used to link diary entries together and to send automated reminder emails about diary completion during the study period. As a participation incentive, all parents were invited to enter a prize draw for €50 gift vouchers.

### Pre-diary Questionnaires

Parents completed two measures prior to completing their first diary entry. First, parents completed the *Inventory of Parent/Caregiver Responses to the Children’s Pain Experience (IRPEDNA)*, measuring their typical behaviors when their child is in pain (Huguet et al., 2008). Next, parents completed the *Parental Catastrophizing Scale-Parent Version* (PCS-P; Goubert et al., 2006). Completion of these measures took approximately 5–10 min.

### Diary

As everyday pain events occur frequently but spontaneously, a reporting period of fourteen consecutive days was considered sufficient. Parents completed one electronic diary entry at the end of each day, describing one pain event that their child had experienced that day (Figure 1B). Where multiple pain events occurred in a single day, parents were asked to report the most “memorable” event. When parents clicked the diary survey link, an information screen explained how to complete the survey, and advised parents that they could skip any question they did not wish to answer. This introduction screen also listed contact details for the lead researcher in case parents had queries or wanted to withdraw participation. The second screen contained the diary questions, with a progress bar along the top of the screen. The third and final screen confirmed their submission, greeting parents with a “Thank you” message, and a reminder to complete another diary entry the following day. The page would automatically refresh back to the introduction screen if not closed by the parent. Completion of the diary would take approximately 5 min each time.

### Ecological Momentary Assessment

As the diary was completed solely by parents, an ecological momentary assessment (EMA) was incorporated to explore the child’s perspective of everyday pains. Parent-child pairs were invited to complete at least one EMA assessment together during the same fourteen-day period that parents completed the end-of-day diaries. Parents were instructed that the EMA questions should be completed on their smartphone *immediately* after a chosen pain event took place, rather than at the end of the day (Figure 1C). Completion took approximately 2–3 min.

## Materials

### Pre-diary Questionnaires

#### IRPEDNA

Parents first completed the *IRPEDNA* (Huguet et al., 2008), a 37-item inventory listing a parent’s typical behaviors when their child experiences pain. The three sub-scales, *Solicitousness, Discouragement*, and *Promotion of Well-behaviors and Coping* present self-oriented statements (e.g., “*I use humor to take his/her mind off the discomfort*”), scored on a scale of 0–5 (0 = “*Never”* to 5 = “*Always”*). Scores are calculated by averaging the items for each subscale, with higher scores indicating higher levels of the respective behavior. For this study, two items from the Solicitousness subscale were removed, as they were not relevant for pre-school children: (1) *“I accept that, in these circumstances, he/she need not do his/her homework”*, and (2) *“I tell his/her teachers how he/she is feeling so that they are aware of the problem during school hours.”* Two further items had their wording amended, to make them applicable to younger children: (1) “I help him/her to do certain things, i.e., *get dressed, do homework*” (words in italics were removed from the original item); and (2) “I try to get him/her to be optimistic about the pain, i.e., *I told them the pain will go away soon*” (words in italics were added to the original item). Scores were calculated as described above, minus the two omitted items. The IRPEDNA has been validated for parents of school-age children, but to our knowledge, has not been used in parents of younger children; thus, a reliability analysis was conducted on our sample. The Cronbach’s alpha for all subscales was excellent (Solicitousness, α = 0.89; Discouragement, α = 0.91; and Coping-Promoting, α = 0.88).

#### Parental Catastrophizing Scale-Parent

Next, parents completed the *PCS-P* (Goubert et al., 2006), a 13-item scale describing catastrophic thoughts and feelings that parents may have about their child’s pain, divided into three sub-scales: *Rumination, Magnification*, and *Helplessness*. Each item was rated on a six-point scale (0 = “*Not at all”* to 5 = “*Extremely”*) and scores were calculated by averaging the subscale item scores, with higher scores indicating higher levels of catastrophizing (Goubert et al., 2006). This scale demonstrates good validity in parents of children of different ages (Goubert et al., 2006; Caes et al., 2012), but was originally validated in parents of school-aged children (9–16 years) and of children with chronic pain and has not previously been tested in younger cohorts nor those experiencing milder pains. The Cronbach’s alpha for the PCS-P scale in this sample showed excellent reliability, α = 0.90.

#### End-of-Day Diary

The end-of-day pain diary contained twenty-three items (Supplementary Data Sheet 1). Parents first confirmed if their child had experienced a pain event that day and stated the number of pain events that had occurred. If no pain events occurred, parents were not required to complete the remaining questions and could submit that diary entry immediately. All remaining questions in the diary asked the parent to answer in relation to whichever they felt had been the “*most memorable pain event”* of that day (if more than one event had occurred). The following questions were derived from validated reporting measures of child pain:

A **pain manikin** (modified from Noel et al., 2018) visually indicated the bodily location of the pain. This modified version included numbered body parts, to allow parents to indicate the numeral associated with the affected body part. If none of the numbered body parts matched the location of their child’s pain, parents could manually type in the area.

Next, parents rated the level of pain intensity they felt their child had experienced using the Faces Pain Scale-Revised (FPS-R) (Hicks et al., 2001). This scale comprised six faces showing increasing amounts of pain, scored from 0 to 10 in 2-point intervals, with higher scores indicating more pain. As one of the leading scales for pediatric self-report, the FPS-R has been validated extensively in preschool populations and can be administered without training, making it suitable for parents (Hicks et al., 2001; von Baeyer et al., 2017).

Two items were included from the Dalhousie Everyday Pain Scale (DEPS) (Fearon et al., 1996) to assess parents’ estimation of their child’s *“pain severity”* and “*intensity of child distress”* during the chosen pain event. Both items were recorded on ordinal scales, with higher scores indicating higher pain severity (0 = “*No hurt”* to 4 = “*Severe hurt”*) and child distress (0 = “*No distress”* to 5 = “*Screaming”*), respectively. The DEPS exhibits strong validity and inter-rater reliability for 3–7-year-olds, having been used in multiple previous studies which examined everyday pain experiences in preschool children (Fearon et al., 1996; Noel et al., 2018; O’Sullivan et al., 2019).

To give insight into parent behaviors during the pain events, and to explore whether daily behaviors correlated with parental perceptions about their behaviors prior to participating, seven items from the **IRPEDNA** (Huguet et al., 2008) were included: three from the Solicitousness subscale, one from the Discouragement subscale, and three from the Coping-Promoting and Wellbeing subscale. The items chosen were those with the highest factor loading on each subscale. For the end-of-day diary, all items were rephrased into past tense (i.e., “When my child *was* in pain…”) to reflect that parents were reporting on a past event, and each item was rated on a five-point scale (0 = “*Not at all”* to 4 = “*Extremely”*). Mean scores were calculated by averaging the items for each subscale. The Cronbach’s alpha scores were acceptable: Solicitousness subscale, α = 0.79; Coping-Promoting subscale, α = 0.71. An alpha-score for Discouragement could not be computed, as only one item was included.

The three-item **State Pain Catastrophizing Scale-Parent version** (Durand et al., 2017) comprises the item with the highest factor loading from each respective subscale (Rumination, Magnification, and Helplessness). In this study, the *PCS-P State* was used to assess state-level catastrophic thoughts specifically related to the pain event that occurred that day and explore whether parental reported feelings correlated with perceptions about their feelings prior to participating (see *Pre-diary Questionnaires*, above). Each statement was rephrased into past tense (i.e., “I *stopped* what I was doing…”) and each item was rated on a five-point scale (0 = “*Not at all”* to 4 = *”Extremely”*). Mean scores were calculated by averaging the item scores. The Cronbach’s alpha for the diary PCS-P was acceptable; α = 0.70.

The four-item distress subscale from the **Parental Distress and Sympathy Questionnaire (PDSQ)** was included (Caes et al., 2011). The diary version of the scale comprised four self-oriented statements of distress, “I felt __________ (worried/upset/anxious/sad).” All statements were rated on an 11-point scale (0 = “*Not at all”* to 10 = “*Extremely”*). Mean distress scores were calculated by averaging the item scores. The Cronbach’s alpha for the PDSQ was excellent; α = 0.87. The use of emotional adjectives to measure parental emotions has proven to be a reliable method and has previously been validated in parents of young children (Goubert et al., 2008; Caes et al., 2011).

The remaining three questions were designed specifically for this study: first, drawing from relevant work on childhood injuries (Morrongiello et al., 2004), parents were asked “*At the time of the pain event, were you nearby?*” This measure contained five decreasing levels of supervision, with higher scores indicating lower levels of parental supervision (Q8, Supplementary Data Sheet 1). Next, two questions were open-ended, allowing parents to provide a description of the context surrounding the event (Q3, Supplementary Data Sheet 1), and include any additional details which they felt were relevant (Q10, Supplementary Data Sheet 1). This would offer insight into which details parents considered “memorable” from their child’s pain events.

#### Ecological Momentary Assessments

The EMA was completed by child and parent on the parent’s smartphone, immediately after any pain event of their choosing (Supplementary Data Sheet 2). Parents were instructed to ask their child to confirm (“*Yes/No*”) whether they were currently experiencing pain. If “Yes,” the child answered the second question (“*How much pain?*”) using the *Simplified Faces Pain Scale (S-FPS)* (Emmott et al., 2017). This three-point visual scale shows three faces of increasing pain or discomfort (*0*, *No hurt; 1*, *Some hurt; 2*, *A lot of hurt*). If the child indicated “No” to the first question, they were not asked the second question. The S-FPS has been validated for use with 3–4-year-olds (Emmott et al., 2017). Next, the parent gave a proxy estimate for their child’s pain, also using the S-FPS, and completed the four-item distress subscale of the Parental Distress and Sympathy Questionnaire (Caes et al., 2011) (see section “Materials” and section “End-of-day Diary”). While the original PDSQ scale has been tested using either an 8-point or 11-point rating scale, the EMA in this study used a modified 6-point scale (0, *“Not at all”* to 5, *”Extremely”*) to improve usability as a shorter scale was easier to complete on narrow smartphone screens (Figure 1D). The Cronbach’s alpha for the modified PDSQ was excellent; α = 0.86. Mean distress scores were calculated as above (Caes et al., 2011).

### Data Analysis

Demographic data and quantitative responses were analyzed using descriptive tests in SPSS 24.0 (IBM Corp, Armonk, NY, United States). Statistical significance for all tests was set at α level of *p* < 0.05. Sex effects, supervision, and EMA ratings were compared using independent *t*-tests, while age effects were compared using one-way ANOVA (four groups: 2, 3, 4, and 5-year-olds), followed by *post hoc* (LSD) tests where appropriate. Spearman’s correlations were used to detect relationships between parental distress and their pain estimates in the EMA. Hierarchal level modeling (HLM) was used to analyze the diary data. A series of five hierarchical regression analyses were conducted using HLM 8.0 (Scientific Software International, Inc., Skokie, IL, United States). Maximum likelihood estimation was used for all models, which consisted of data from daily diary entries (level 1; *N* = 151), nested within parental characteristics (level 2; *N* = 21). This is considered an adequate sample size for obtaining reliable parameter estimates (Snijders and Bosker, 2011). Missing data was excluded by the model prior to analysis. Level 1 and level 2 predictors were each grand mean centered. For each model, analyses were conducted in three phases: unconstrained (null) model, random intercepts model, and means-as-outcomes model. The 5 models explored the contribution of contextual factors on the parent’s daily responses to their child’s everyday pain experience: (1) Parental distress, (2) Parental solicitousness, (3) Parental discouraging, (4) Parental coping-promoting, and (5) Parental catastrophizing. At level one, parental supervision, mean state (daily) parental catastrophizing, and child pain intensity, severity and distress were entered. At level two, child sex, child age, parent age, and mean trait (pre-diary) parental catastrophizing were entered. Additionally, when analyzing the contribution of trait-level responses from the IRPEDNA and PCS-P, the trait scores from these measures were also entered at level 2.

## Results

### Description of the Typical Daily Pain Events

The average number of completed diary entries per family was *M* = 8.52 entries (range: 1–19). A total of 197 end-of-day diary entries were completed: 157 entries (79.7%) described a pain event, while 40 diary entries (20.3%) reported no pain events for that day. The total number of pain events ranged from 0 to 6 incidents each day (*M* = 1.83; *SD* = 1.33).

Parents reported crying as the predominant child response to pain events (*N* = 71; 46.1%), while a large number of children also made verbal comments (e.g., “Ouch!”) (*N* = 38; 24.7%). Other behaviors included sobbing (*N* = 23; 11.7%), screaming (*N* = 10; 6.5%), and facial expression (*N* = 10; 6.5%). In only two incidents, the child gave no sign of distress (1.3%). Parents generally estimated that pain events were of low pain severity (*M* = 1.65, *SD* = 0.89), with most events rated as mild (*N* = 64; 40.8%) or moderate severity (*N* = 56; 35.7%). Parents estimated one-third of all everyday events (*N* = 54; 34.4%) as mild or moderate intensity, while approximately half of pain events (*N* = 81; 51.6%) were scored as severe or extreme intensity, leaving an average parental estimate of “moderate” pain intensity (*M* = 4.20, *SD* = 1.5).

The sites of injury were varied. Over one-third of all reported incidents involved an injury to the child’s head (*N* = 58; 36.9%), with the next most frequent sites of injury being hands (*N* = 24; 15.3%) and knees (*N* = 20; 12.7%) (Table 2).

**TABLE 2:** Breakdown of the reported sites of injury in “everyday” pain events.

Site of injury	Number of reported incidents	Percentage (%)
Head	58	36.9
Hand	24	15.3
Knee	20	12.7
Foot	14	8.9
Back	9	5.7
Leg	9	5.7
Buttocks	7	4.5
Elbow	5	3.2
Shoulder	4	2.5
Multiple sites	3	1.9
Stomach	2	1.3
Other/Do not know	2	1.2

### Parental Emotional and Behavioral Responses During Daily Pain Events

In the pre-diary questionnaires, in response to a hypothetical daily pain event that their child may experience, parents recorded low anticipated levels of Solicitousness (*M* = 2.52, *SD* = 0.52), Discouragement (*M* = 1.48, *SD* = 1.25), and Coping-Promoting behaviors (*M* = 2.59, *SD* = 0.69), and low anticipated levels of catastrophizing thoughts (*M* = 1.52, *SD* = 0.83).

In completing the daily pain diary, parents reported low levels of Solicitousness (*M* = 1.55, *SD* = 1.09), Discouragement (*M* = 0.38, *SD* = 0.81), and Coping-Promoting behaviors (*M* = 1.55, *SD* = 1.12) in response to their child’s daily experienced pain events. Parents also reported low daily levels of catastrophizing thoughts (*M* = 0.86, *SD* = 0.74) and gave low ratings for their own distress (*M* = 1.60; *SD* = 1.71) in response to their child’s pain event: out of 156 pain events, parents rated 140 events (89.7%) as causing a low level of distress (i.e., a score of 5 or below on the 11-point scale).

### Influence of Parental Supervision

**FIGURE 2:**
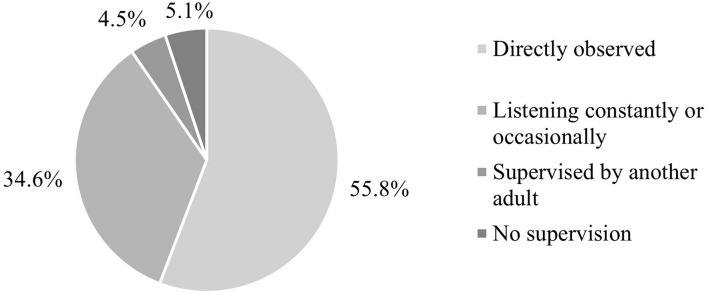
Levels of parental supervision.

In just over half of events (*N* = 87, 55.8%), parents had directly observed or witnessed their child’s pain event incident, while in a further 54 incidents (34.6%), parents had been listening in either constantly or occasionally. Only a small proportion of events were not observed by the parent. As such, the original five categories were collapsed into two levels for analysis: “Present” (*directly observed the event*) or “Not present” (all other levels: *listening in constantly or occasionally, supervised by another adult, no supervision*) (Figure 2).

Pain events occurred less frequently when parents were present for their child’s pain event (*M* = 1.84, *SD* = 1.08) compared to parents who were not present (*M* = 2.26, *SD* = 1.45); *t*(154) = 2.11, *p* = 0.037. Parents who were present for pain events reported significantly lower levels of personal distress (*M* = 5.02, *SD* = 5.96) compared to parents who were not present (*M* = 8.39, *SD* = 7.62); *t*(153) = 3.09; *p* = 0.002. Parental catastrophizing was significantly elevated if parents had not been present for pain events (*M* = 3.11, *SD* = 2.44) compared to parents who had been present (*M* = 2.20, *SD* = 1.99); *t*(153) = −2.55, *p* = 0.012. There was no significant association between parental supervision and estimates of child distress, pain severity, or pain intensity; all *p* > 0.05.

### Influence of Child Sex and Age on Parental Pain Estimates

Child sex influenced how parents estimated their child’s pain experiences. Parents gave significantly higher pain estimates of pain intensity for girls (*M* = 4.81, *SD* = 1.36) than for boys (*M* = 3.84, *SD* = 1.47); *t*(155) = 4.12, *p* < 0.001. Though parents of boys reported 109 pain events (*M* = 1.76, *SD* = 1.23) and parents of girls reported 62 pain events (*M* = 1.98, *SD* = 1.48), this difference was not significant, *t*(169) = 1.06; *p* > 0.05. Child sex did not influence parent estimates of child distress, pain severity, or parental supervision; all *p* > 0.05.

Child age influenced parent estimates of child pain intensity, *F*(3,153) = 4.01, *p* = 0.009. *Post hoc* tests revealed that parents gave significantly lower estimates for 5-year-olds (*M* = 3.46, *SD* = 1.44; *N* = 22) than for 2-year-olds (*M* = 4.88, *SD* = 1.33, *N* = 24; *p* = 0.001) and 3-year-olds (*M* = 4.36, *SD* = 1.45, *N* = 50; *p* = 0.016), but not 4-year-olds; *p* > 0.05. Child age also influenced parent estimates of child distress, *F*(3,150) = 2.91, *p* = 0.036: parents gave significantly lower estimates for 5-year-olds (*M* = 2.70, *SD* = 0.73, *N* = 20) than for 3-year-olds (*M* = 3.50, *SD* = 1.07, *N* = 50; *p* = 0.008), but not 2 or 4-year-olds; both *p* > 0.05. Child age did not influence number of pain events, parental supervision, or parent estimates of pain severity; all *p* > 0.05.

### Influence of Contextual Factors on Parental Responses

A series of five hierarchal linear regression analyses were conducted on the influence of contextual factors in contributing to parental daily responses to their child’s pain (i.e., parental distress, solicitousness, discouragement, coping-promotion, and catastrophic thinking, Table 3). The analysis with parental daily distress as an independent variable returned an ICC of 0.432 (43.2% of the variance was between parents, and 56.8% was within parents). Parent distress was significantly associated with pain severity (β = 0.34, *p* = 0.013, *r* = 0.16), and daily catastrophizing (β = 1.28, *p* < 0.001, *r* = 0.45). Parent distress levels were increased in those who had interpreted their child’s pain event as more severe, and in parents who had reported increased levels of state-level catastrophic thinking (Table 4).

**TABLE 3:** Mean, SD, and range of contextual factors for HLM analyses.

Variable	*N*	Mean	*SD*	Range
**LEVEL 1**				
Mean (daily) catastrophizing	151	0.84	0.72	0–3.67
Mean IRPEDNA score				
*Solicitousness*	151	1.55	1.09	0–4
*Discouragement*	151	0.38	0.82	0–4
*Coping promotion*	151	1.55	1.11	0–4
Parental distress	151	1.54	1.64	0–6.25
Pain severity	151	1.64	0.87	0–4
Pain intensity	151	4.21	1.48	1–6
Estimates of child distress	151	3.17	1.14	0–5
**LEVEL 2**				
Mean (pre-diary) catastrophizing	21	1.52	0.83	0.33–3.67
Mean (pre-diary) IRPEDNA score				
*Solicitousness*	21	2.52	0.52	1.67–3.67
*Discouragement*	21	1.48	1.25	0–4
*Coping promotion*	21	2.59	0.69	1.33–3.67

**TABLE 4:** Final models for influence of contextual factors on parental responses.

	Parental daily Distress			Parental daily solicitousness			Parental daily discouragement			Parental daily coping-promoting			Parental daily catastrophizing		
Variable	β coeff.	SE	*T*	β coeff.	SE	*T*	β coeff.	SE	*T*	β coeff.	SE	*T*	β coeff.	SE	*T*
Intercept (β0)	1.64	0.19	8.49	1.60	0.11	14.87***	0.47	0.11	4.33***	1.70	0.14	12.40***	0.81	0.08	10.42***
Child age (γ01)	–0.03	0.22	–0.15	–0.26	0.13	−2.07#	–0.29	0.13	−2.31*	–0.25	0.16	–1.59	–0.07	0.09	–0.79
Parent age (γ02)	–0.03	0.05	–0.67	–0.03	0.03	–0.90	–0.03	0.03	–1.13	–0.03	0.04	–0.82	–0.05	0.02	−2.43*
Child sex (γ03)	–0.40	0.42	–0.96	0.58	0.24	2.41*	0.10	0.24	0.42	–0.03	0.30	0.09	–0.19	0.17	–1.16
Mean (pre-diary) PCSP-State (γ05)	0.24	0.26	0.94	–0.20	0.15	–1.30	0.04	0.14	0.26	–0.04	0.18	–0.21	–0.04	0.10	–0.37
Mean (pre-diary) IRPEDNA score (γ05)	−	−	−	0.50	0.27	1.88#	0.05	0.11	0.51	0.50	0.24	2.09#	−	−	−
Pain severity (γ10)	0.34	0.14	2.51*	0.06	0.11	0.51	–0.17	0.10	−1.69#	–0.15	0.11	–1.41	0.44	0.07	6.70***
Pain intensity (γ20)	–0.02	0.10	–0.17	–0.05	0.08	–0.68	–0.04	0.07	–0.50	0.09	0.08	1.23	–0.04	0.05	–0.75
Estimates of child distress (γ30)	0.08	0.11	0.69	0.24	0.09	2.61**	0.03	0.08	0.32	0.09	0.09	1.09	0.11	0.06	1.88#
Parental supervision (γ40)	–0.07	0.14	–0.52	–0.02	0.11	–0.16	–0.15	0.10	–1.44	–0.01	0.11	–0.11	–0.09	0.08	–1.16
Mean (daily) PCSP-State (γ50)	1.28	0.15	8.53***	0.67	0.12	5.65***	–0.13	0.11	–1.16	0.55	0.12	4.73***	−	−	−

Coeff., coefficient; *p < 0.05; **p < 0.01; and ***p < 0.001.

*#*p*-value was borderline of significance (*p* = 0.051–0.09).*

*Significant values are indicated in bold.*

Parental daily solicitousness returned an ICC of 0.415 (41.5% of the variance was between parents, and 58.5% was within parents). Parent solicitousness was significantly associated with child sex (β = 0.58, *p* < 0.05, *r* = 0.26), child distress (β = 0.23, *p* = 0.01, *r* = 0.17), and daily catastrophizing (β = 0.67, *p* < 0.001, *r* = 0.34). Parental solicitousness was increased in parents of girls, when parents interpret their child as being more distressed, and when parents report higher levels of state-level catastrophic thinking.

Parental daily discouragement returned an ICC of 0.586 (58.6% of the variance was between parents, and 41.4% was within parents). Discouragement was significantly associated with child age (β = −0.29, *p* < 0.05, *r* = 0.27), indicating decreased parental discouragement with increasing child age.

Parental daily coping-promotion returned an ICC of 0.590 (59% of the variance was between parents, and 41% was within parents). Parent daily (state) coping-promotion was significantly associated with daily catastrophizing (β = 0.55, *p* < 0.001, *r* = 0.25), revealing increased coping-promotion when parents reported higher levels of state-level catastrophic thinking.

In the final model, parental daily catastrophizing returned an ICC of 0.287 (28.7% of the variance was between parents, and 71.3% was within parents). Catastrophizing was significantly associated with pain severity (β = 0.44, *p* < 0.001, *r* = 0.43) and parent age (β = −0.05, *p* < 0.05, *r* = 0.28). Increased levels of catastrophic thinking were reported when parents interpreted the event as more severe and by younger parents.

### Ecological Momentary Assessments

In total, 47 EMA entries were collected from 9 parent-child pairs (42.9% of parents completing end-of-day diary entries during the same period), with pairs completing on average one EMA entry per day (range: 0–3 per day). There was a significant difference between parent and child pain estimates; t(77) = 3.83; *p* < 0.001, with children reporting higher pain estimates (*M* = 2.43; *SD* = 0.77; *N* = 37) compared to their parents (*M* = 1.88; *SD* = 0.59; *N* = 42). Parents who indicated that their child had experienced a high level of pain or hurt gave higher ratings for their own distress than parents whose child experienced a low level of pain or hurt: *r*_*s*_ (46) = 0.46, *p* < 0.001. Parents of 3-year-olds submitted the most entries (*N* = 27, 57.5%), followed by parents of 2-year-olds (*N* = 14, 29.8%), and 4-year-olds (*N* = 6, 12.7%); no entries were submitted by parents of 5-year-olds.

## Discussion

This study investigated the context surrounding parental responses to minor everyday childhood pain experiences, through the diary records of parents as the primary “witnesses” to these experiences, and the novel use of electronic momentary assessment (EMA) to compare parent and child ratings of everyday pain events. The pain diary methodology allowed for a clearer appreciation of the context surrounding everyday pain events, as parents provided their own observations on these events and gave insights into their child’s experience of pain. The EMA provided valuable insight into the child’s own experience of pain in comparison to parental estimates. However, it is unclear whether the EMA was of interest to parents and a feasible method on a large scale as uptake was low, and most parents typically only completed the EMA once per day. Additional larger-scale studies are needed to explore the usability of EMA in capturing everyday pain events. The diary incorporated items from existing scales, including the Dalhousie Everyday Pain Scale (DEPS). The DEPS has previously been utilized by researchers in a range of natural environments, including day-care, play centers, and at home (Fearon et al., 1996; Noel et al., 2018; O’Sullivan et al., 2019). Within this study, the DEPS was used solely by parents at home, yet the reporting is consistent with previous literature. For example, the most-reported sites of pain (e.g., head, hands, and knees) were similar to those reported in a day-care environment (Fearon et al., 1996), and similar estimates of pain severity were given by the parents in this study and by parents observing their child’s pain events in an activity center (e.g., approximately 75% of incidents in both settings were considered low severity) (Noel et al., 2018). This suggests that minor pain events were similar across settings, and that the DEPS can be used to adequately capture pain events in the home through parental daily reporting.

In the present study, the DEPS revealed a notable disparity in parent estimations of pain situations: while parents rated most incidents as low severity, they simultaneously indicated that the child was demonstrating severe or extreme pain intensity. This might indicate that parents are aware that differences exist between their *observations* of the incident and their child’s *experience* of the incident. For example, the parent observes a seemingly minor pain incident, but their child exhibits exaggerated behaviors (“tantrums”) which seem incongruous with the severity of the incident. However, the parent recognizes that their child may be frustrated or upset but cannot communicate their comfort needs in that moment; thus, the parent interprets their child’s behaviors as part of their experience which differs from their own observation. This supports recommendations that contextual observation by clinicians and caregivers should be complemented with self-report from the child where possible (while acknowledging their limited reliability among very young children) (von Baeyer, 2009). This recommendation seems to be further confirmed by the data captured through EMA, which revealed that parental estimates of pain intensity were consistently lower than the child’s own estimates. These findings echo those from clinical settings, wherein parent proxy ratings have continually been shown to underestimate pain compared to the child’s self-report (Manne et al., 1992), and highlights that incongruous pain ratings also extend beyond clinical settings. While pain intensity is not typically informative where pain is already anticipated to be strong (i.e., post-surgery), it is relevant to caregivers who manage acute pain events regularly. Gathering pain intensity ratings from the child, using validated but simple assessments such as the S-FPS, may allow caregivers to assess pain quickly and determine whether further treatment is required (Cohen et al., 2019). Future studies could consider whether additional measures can provide a reliable means of drawing young children into conversations about their own pain. For example, a body outline tool was used by the parents in this study, and similar tools have been used previously by older children and adolescents (Savedra et al., 1989) but have not yet been validated in younger children (Mesko and Clark, 2019). Equally, the use of coloring tools could allow preschool children to report differing levels of pain (Mahon et al., 2015).

Our findings also revealed that parent’s interpretations of everyday pain experiences were moderated by child sex. Parents of girls gave much higher estimates of their child’s pain intensity than parents of boys. These findings contrast against recent literature which indicated that adults rate pain more highly in boys than in girls (Cohen et al., 2014; Earp et al., 2019). However, sex differences in adult ratings are widely inconsistent, with evidence that parents often over- and underestimate girls’ pain (Schinkel et al., 2018), or give equivalent ratings for boys’ and girls’ pain (Goodenough et al., 1999). There is extensive evidence that sex differences are rarely found in *children’s own estimates* of pain intensity, tolerance, or affect (Boerner et al., 2014), suggesting that adult ratings may be biased by gender stereotypes regarding pain behaviors in boys versus girls (Earp et al., 2019). Adult responses to child pain also vary based on their own sex: fathers gave higher pain ratings to their sons than their daughters, while mothers ratings did not differ (Moon et al., 2008). It was not possible to conduct a similar analysis in the current study due to the disproportionately female sample (95% mothers). Pediatric pain studies predominantly feature data from only the child’s mother, and challenges with recruiting fathers into studies to explore parental responses to child pain have been highlighted previously (O’Sullivan et al., 2021). Given our own findings, we propose that sex differences in parental ratings may also vary depending on the *type of pain*, as the listed studies examined medical or experimental procedures while the present study captured everyday pains. Previous studies of everyday pains have demonstrated that girls exhibited higher personal control and were often playing alone prior to pain events (O’Sullivan et al., 2019), but exhibited more visible and vocal distress during pain events (Fearon et al., 1996). Thus, while pain events may be of lower impact amongst girls due to a more reserved play style, their responses may induce adults to rate pain events as more intense for girls.

In exploring the role of influencing factors on parental responses toward their child’s everyday pain experiences, our findings revealed that parental trait levels or expectations of their behaviors and catastrophizing thoughts during their child’s pain experiences (taken from their pre-diary questionnaire responses) did not always correlate with their actual or state responses (reported in the daily diaries). Instead, contextual factors (e.g., estimated child pain severity or distress) and child characteristics (e.g., sex and age) were typically stronger factors influencing parental feelings, thoughts, and behaviors. For instance, in line with previous evidence for pediatric chronic pain, parental estimates of their child’s pain severity and distress was related to increased levels of their own distress and solicitousness (Langer et al., 2014). Child sex was a significant influence on parental solicitousness during everyday pains; this expands findings from chronic pain literature that parents respond more protectively toward girls than boys (Langer et al., 2014), by confirming that child sex also influences parental responses to other types of child pain. Taken together, these findings extend the evidence that parent (trait) expectations of their thoughts and behaviors do not necessarily match their (state-dependent) behaviors during their child’s pain in real life (Durand et al., 2017), thereby highlighting the necessity to capture parental situation-specific responses.

State (daily) levels of catastrophic thinking, in particular, represented a significant influence on how parents responded during pain events, as parental levels of distress, solicitousness, and coping-promoting were all increased when parents reported increased catastrophizing thoughts for that particular pain event. This echoes previous findings from clinical and experimental pain situations (Caes et al., 2011; Langer et al., 2014), and also extends our understanding of pain catastrophizing beyond these settings by demonstrating that catastrophizing is also a potent influence on parental behaviors in everyday pain situations. We further found that parents who reported increased catastrophizing engaged in more coping-promoting behaviors; this is a departure from existing literature which has generally demonstrated that parental catastrophizing is not associated with adaptive behaviors. As the bulk of the prior literature has focused on youth with chronic pain, it is plausible that the everyday pain experiences recorded in this study simply represent a different context to those previously reported, as they occur in the family’s natural environment without the intrusion of researchers or medical professionals. Thus, behaviors demonstrated by parents in everyday pain contexts, such as those reported here, may be a natural or instinctive response of parents to their child’s pain, and these may be more varied and include efforts to encourage or otherwise engage their child in coping-promoting behaviors and teach them to manage pain effectively. However, additional research is required before we can draw sharper insights into the potential differential impact of parental catastrophic thinking on parental behaviors across various pain contexts.

Beyond the influence of parental daily catastrophic thinking, parental level of supervision was key in understanding both the child’s pain experience and parental responses: pain events occurred more frequently, and parents reported higher catastrophizing and personal distress if they had not been actively supervising their child during the pain event. This expands findings from unintentional injury literature, that active supervision is protective against in-home injuries (Morrongiello et al., 2004), while parental distress is associated with increased rates of child injury (Schwebel et al., 2011) and confirms these effects also occur in the context of everyday pains. However, in the present study, level of supervision did not influence how parents responded to their child, or the estimates that parents gave for their child’s pain severity and intensity, or distress. This echoes previous findings that parents rarely change their responses or strategies following an incident, if they felt they were already doing all they could to prevent it (Morrongiello et al., 2004). The design of this study was drawn from longitudinal studies of childhood injuries, which explore supervision in relation to pain events occurring during short, defined periods (e.g., 12 weeks). Future studies could explore supervision within the home over similar periods, to determine whether parental responses to everyday pains change over time (for example, as their child grows older and in less need of supervision).

### Limitations

Attrition rates between recruitment and daily reporting were high: only 58% of recruited parents moved onto diary completion following the demographics stage, and only 43% of parents completing diaries also completed EMA. Interested parents could self-enroll into the study, and due to the nature of the data collection, the only contact point was via email. The non-participating parents were contacted through email to remind them of diary completion and emailed at the end of the study to inquire about reasons for attrition; however, no responses were received. This limits the insight available into why some parents did not proceed to join the study proper. Similar attrition rates have previously been reported among electronic diary users (Gaertner et al., 2004). The final sample was slightly underpowered for this study, and a larger sample could increase the strength of some findings; similar designs should account for this within their recruitment plans. Furthermore, the sample was overly homogenous: 90% of participants were resident in Ireland, and 95% of diary entries were completed by mothers. These factors reduced the ability to extend our findings to other populations. While basic demographic data was collected to support the analyses (age, sex, parental education, and number of children in the household), the collection of further data relating to socioeconomic status, parental mental health, or family environment/support could have added more context to the findings. In particular, the relationships between parental supervision and frequency of pain events, parental distress and catastrophizing could have been enriched by obtaining parental estimates of how often they check on their child (number or hours spent per day in supervising their child) or perceptions about their parenting style (i.e., if they feel they are anxious or “hover” around their child). Finally, selection bias cannot be ruled out as parents may have chosen events that they could more clearly recall, or events which would appear socially acceptable. For example, most incidents were reported when parents were actively supervising, as parents may not have wanted to divulge that they were not monitoring their child. While the diary and EMA prevented us from examining the pain events that were not reported, they give insight into the types of pain events that parents consider “memorable” enough to report. Future studies may benefit from asking parents to report multiple pain events within the same day, to ensure a better representation of pain events within the home.

## Conclusion

The parent pain diary provided a key insight into the nature of “everyday” pain experiences around the family home, without disrupting the natural behavior of their child. Parental responses to their child’s pain are influenced “in the moment” by their judgments of the severity and intensity of the incident. Formal assessments of pain are inappropriate outside of clinical settings, but even within the home, parents naturally engage in levels of investigation when determining how severe an incident may have been, particularly when they themselves did not witness the pain event. Short assessments, such as those described here, may assist parents with gaining information about everyday pain events, and provide opportunities to model adaptive coping behaviors when their child experiences pain.

## Data Availability Statement

The raw data supporting the conclusions of this article will be made available by the authors, without undue reservation.

## Ethics Statement

The studies involving human participants were reviewed and approved by NUI Galway University Research Ethics Committee. Written informed consent to participate in this study was provided by the participants’ legal guardian/next of kin.

## Author Contributions

GO’S led the study design, participant recruitment, collection, analysis, and interpretation of the data, with input on data analysis from LC, and wrote-up of the manuscript. All authors contributed to the editing of the manuscript, and read and approved the final manuscript.

## Conflict of Interest

The authors declare that the research was conducted in the absence of any commercial or financial relationships that could be construed as a potential conflict of interest.

## Publisher’s Note

All claims expressed in this article are solely those of the authors and do not necessarily represent those of their affiliated organizations, or those of the publisher, the editors and the reviewers. Any product that may be evaluated in this article, or claim that may be made by its manufacturer, is not guaranteed or endorsed by the publisher.
