# Supplementary material for: Exploring Parental Responses to Pre-schoolers’ “Everyday” Pain Experiences Through Electronic Diary and Ecological Momentary Assessment Methodologies
Source: Front Psychol. 2021 Nov 4;12:741963. doi: 10.3389/fpsyg.2021.741963 (PMC8599282; doi:10.3389/fpsyg.2021.741963)
Supplement: Supplementary file 1 [file Data_Sheet_1.docx]

**Daily pain diary**

**Every evening for the next two weeks, we would like you to complete these diary questions about one specific pain event that your child experienced that day (i.e., 14 pain events in total). This should take about 3-5 minutes per evening.**

**If there were no pain events on a given day, or if you missed a day, just fill it in the following day as normal.**

*There are 12 questions in this survey.*

1. **Did your child experience a pain event today:** YES / NO
2. **If yes, how many pain events took place? ___________**

***Please, think of the pain event that stood out the most to you today:***

1. **Can you describe the pain event?**

*For example,*

*“Thomas has two pain events today. Scraped his knee while playing outside. Also bumped his head on the coffee table, and he was crying a bit”*

*“I heard Alice crying from the next room. I didn’t see what happened, but she had a cut on her elbow, so I cuddled her until she calmed down, and then we got a plaster”*

*________________________________________________________________________________________________________________________________________________________________________________________________________________________________________________________________________________________________________________________________________*

1. **For that pain event, please indicate where your child’s hurt/pain was located:**

*(You can circle the location on the picture, or write in the area/zone if that is more convenient)*

**
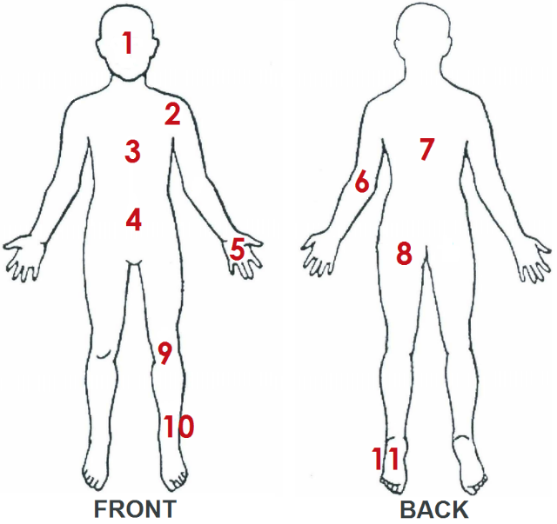
***_____________________________________*

1. **For that pain event, how severe would you rate your child’s hurt? (circle one):**

0 1 2 3 4

NO HURT SEVERE HURT

1. **Choose the face that shows how much your child hurt just after that pain event (circle one):**

**
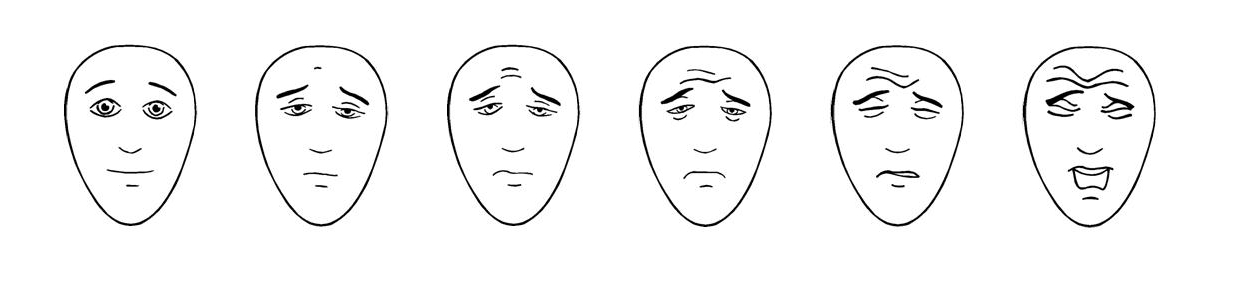
**

1. **For that pain event, what level of distress did your child show when they got hurt? (circle one):**

0 1 2 3 4 5

NONE FACIAL VERBAL SOBBING CRYING SCREAMING

EXPRESSION COMMENT

1. **At the time of the pain event, were you nearby? (circle one):**
   1. *Yes, your child was in view (i.e., you were watching when he/she got hurt and saw the event)*
   2. *No, your child was not in view but you were listening in*
      1. If so, were you listening in constantly or intermittently? (circle one):

*CONSTANTLY / INTERMITTENTLY*

- 1. *No, you did not see or hear the event but another adult was present and saw the event*
  2. *No, you did not see or hear the event but you had been checking on your child before that*

1. **For that pain event, please indicate which answer most closely matches the response you had towards your child (circle one answer for each statement):**
2. *When my child was in pain, I kept thinking about the pain he/she was experiencing*

NOT AT ALL MILDLY MODERATELY SEVERELY EXTREMELY

1. *When my child was in pain, I thought something serious might happen to him/her because of it*

NOT AT ALL MILDLY MODERATELY SEVERELY EXTREMELY

1. *When my child was in pain, I couldn’t stand it any longer*

NOT AT ALL MILDLY MODERATELY SEVERELY EXTREMELY

1. *I stopped what I was doing to do something that my child likes (e.g. play)*

NOT AT ALL MILDLY MODERATELY SEVERELY EXTREMELY

1. *I tried to make up for my child’s suffering by paying him/her more attention*

NOT AT ALL MILDLY MODERATELY SEVERELY EXTREMELY

1. *I monitored their pain afterwards by regularly asking my child how he/she felt*

NOT AT ALL MILDLY MODERATELY SEVERELY EXTREMELY

1. *I made light of the problem because I thought my child was exaggerating a little*

NOT AT ALL MILDLY MODERATELY SEVERELY EXTREMELY

1. *I tried to entertain my child so that he/she did not think about the problem*

NOT AT ALL MILDLY MODERATELY SEVERELY EXTREMELY

1. *I advised my child to relax and breathe deeply*

NOT AT ALL MILDLY MODERATELY SEVERELY EXTREMELY

1. *I told my child that I think everything will be all right*

NOT AT ALL MILDLY MODERATELY SEVERELY EXTREMELY

---------------------------------------------------------------------------------------------------------------------------

*I felt worried*

0 1 2 3 4 5 6 7 8 9 10

NOT AT ALL EXTREMELY

*I felt upset*

1. 1 2 3 4 5 6 7 8 9 10

NOT AT ALL EXTREMELY

*I felt anxious*

0 1 2 3 4 5 6 7 8 9 10

NOT AT ALL EXTREMELY

*I felt sad*

0 1 2 3 4 5 6 7 8 9 10

NOT AT ALL EXTREMELY

1. **Is there anything else that you want to share about today’s pain incidents?**

*________________________________________________________________________________________________________________________________________________________________________________________________________________________________________________________________________________________________________________________________________*
