# Supplementary material for: Exploring Parental Responses to Pre-schoolers’ “Everyday” Pain Experiences Through Electronic Diary and Ecological Momentary Assessment Methodologies
Source: Front Psychol. 2021 Nov 4;12:741963. doi: 10.3389/fpsyg.2021.741963 (PMC8599282; doi:10.3389/fpsyg.2021.741963)
Supplement: Supplementary file 2 [file Data_Sheet_2.docx]

My child just got hurt. What now?

**This short survey looks at how you and your child rate everyday pain events as they happen.**

For this, we would like families to complete this short survey as soon as possible following a pain event that their child has just experienced

If possible, we'd like you to complete this survey up to five times (but if you can only complete it 1-2 times, that's still great and will help a lot!)

**WHAT TO DO:**

If your child has just had one of those "everyday" pain events that often occur (like bumps, scrapes, cuts, etc.), first soothe your child and make sure they're okay.

When they're ready, your child should answer the first two questions on the next page, and then you should answer the remaining three questions.

***[This will take approximately 2-3 minutes to complete]***

*At the end of this study, there will be a prize draw for one of ten €50 One4All vouchers. This draw is open to all families who completed at least 4 out of 5 pain assessments.*

**1. Ask your child: "Are you sore/in pain right now?" (Circle one)**

If your child answers **YES**, ask them the next question with the face picture.

If your child answers **NO**, you don't have to ask them the next question (If they want to answer it anyway, that's fine)


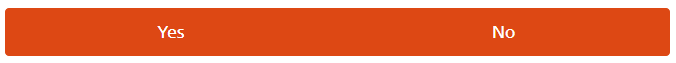

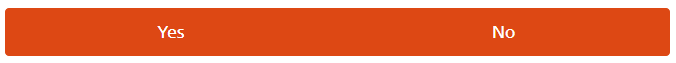


______________________________________________________________________________________

2. CHILD estimate of pain:

These faces show how much something can hurt. This face *(point to the left-most face)*shows no pain/hurt. The faces show more and more pain/hurt up to this one *(point to right-most face)* – it shows a lot of pain/hurt.

Ask your child to indicate which face matches the pain they felt just now.


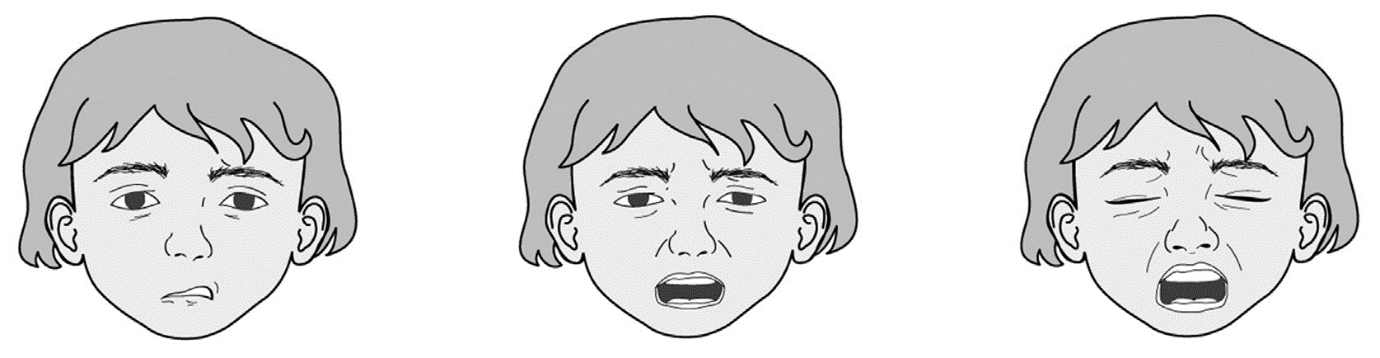


**Face 1 (no pain or hurt) Face 2 (some pain or hurt) Face 3 (a lot of pain or hurt)**

______________________________________________________________________________________

**3. PARENT estimate of pain:**

Please give an estimate for how bad YOU felt your child's pain was just now.


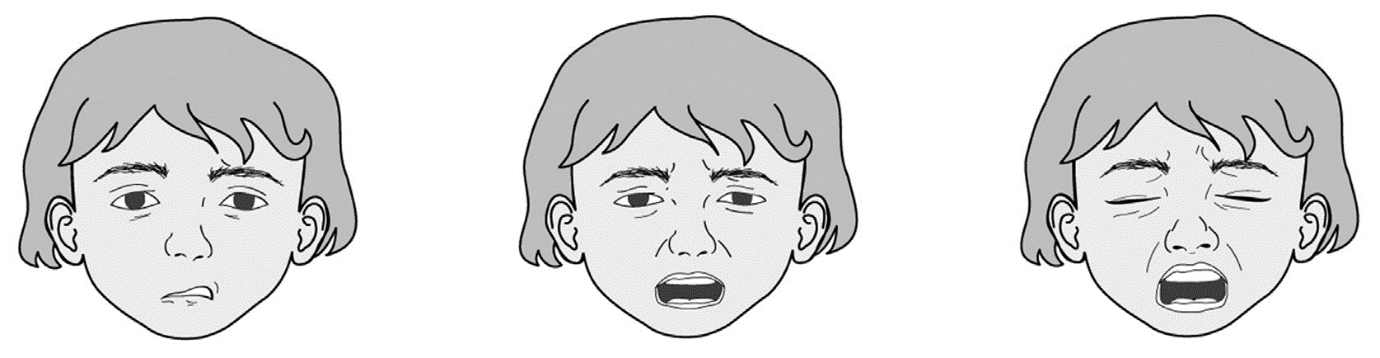


**Face 1 (no pain or hurt) Face 2 (some pain or hurt) Face 3 (a lot of pain or hurt)**

______________________________________________________________________________________

**4. *Parent ratings*:** At this moment, to what extent are you experiencing the following emotions?

(mark one circle for each emotion, where 0= Not at all, and 5= Extremely/a lot):


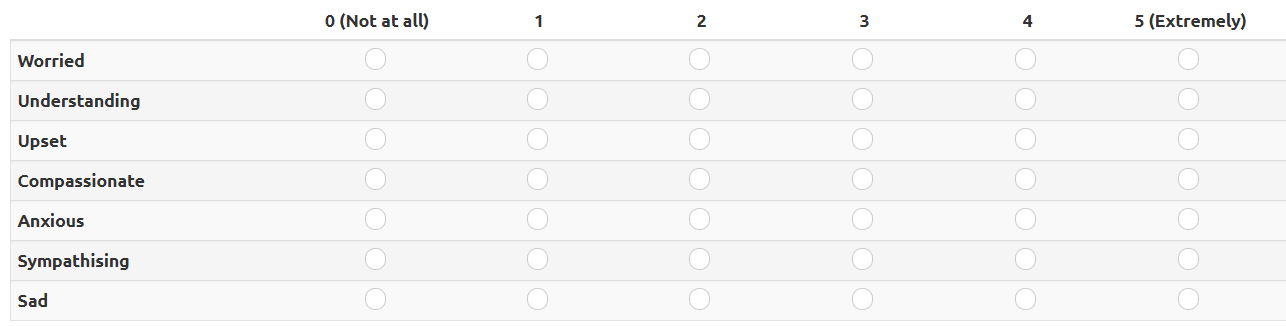


______________________________________________________________________________________

**Thank you!**
